# Microprofiling of nitrogen patches in paddy soil: Analysis of spatiotemporal nutrient heterogeneity at the microscale

**DOI:** 10.1038/srep27064

**Published:** 2016-06-06

**Authors:** Yilin Li, Herbert J. Kronzucker, Weiming Shi

**Affiliations:** 1 State Key Laboratory of Soil and Sustainable Agriculture, Institute of Soil Science, Chinese Academy of Sciences, Nanjing 210008, PR China; 2 Department of Biological Sciences, University of Toronto, 1265 Military Trail, Toronto, Ontario, M1C 1A4, Canada

## Abstract

Flooded paddy soil ecosystems in the tropics support the cultivation of the majority of the world’s leading crop, rice, and nitrogen (N) availability in the paddy-soil rooting zone limits rice production more than any other nutritional factor. Yet, little is known about the dynamic response of paddy soil to N-fertiliser application, in terms of horizontal and vertical patchiness in N distribution and transformation. Here, we present a microscale analysis of the profile of ammonium (NH_4_^+^) and nitrate (NO_3_^−^), nitrification, oxygen (O_2water_ and O_2soil_), and pH (pH_water_ and pH_soil_) in paddy soils, collected from two representative rice-production areas in subtropical China. NH_4_^+^ and NO_3_^−^ exhibited dramatic spatiotemporal profiles within N patches on the microscale. We show that pH_soil_ became constant at 1.0–3.5 mm depth, and O_2soil_ became undetectable at 1.7–4.0 mm. Fertiliser application significantly increased pH, and decreased O_2_, within N patches. Path analysis showed that the factors governing nitrification scaled in the order: pH_water_ > pH_soil_ > NH_4_^+^ > O_2water_ > NO_3_^−^ > O_2soil_. We discuss the soil properties that decide the degree of nutrient patchiness within them and argue that such knowledge is critical to intelligent appraisals of nutrient-use efficiencies in the field.

It is now well established more generally that soil nutrients, including N, are distributed in a heterogeneous or patchy manner within ecosystems^1^,^2^ due to a combination of natural and anthropogenic factors. In agricultural soils, N fertiliser application is the main anthropogenic driver that produces heterogeneity in soil N distribution, and fundamentally affects local N pools and N-cycling processes within soil^3^. Recent research on soil heterogeneity has almost exclusively focused on plant behavior. When roots encounter a nutrient-rich zone or patch, they often proliferate within it, including increases in elongation of individual roots^4^; total root length^5^; root production^6^; and extent of lateral branching^7^. These plastic responses by the root system have been proposed as the major mechanism employed by the root system to allow plants to cope with the heterogeneous supply of nutrients in soil^1^. Variations in both root biomass and N uptake rate per unit root biomass are important in contributing to the variations in the abilities of species to capture N from ephemeral patches^8^. Field studies have shown that plant roots respond most strongly to N given in pulses and least strongly to a continuous nutrient supply^9^. Furthermore, there was a positive relationship between N uptake rate, relative growth rate, and root system biomass^8^. In addition to the plastic responses of plant roots, there have been reports that soil N heterogeneity influences seedling recruitment^10^, vegetation succession^11^, plant species coexistence and competition^12^, and invasion of non-native plants into natural ecosystems^13^.

In flooded paddy soils, N is applied principally as ammonium (NH_4_^+^)-based or urea fertilisers^14^. The use of urea has increased rapidly in the past 40 years, and urea is now the most important N fertiliser in rice production throughout the world^15^. Instead of broadcast fertilisation, urea is frequently applied in a concentrated manner as basal manure, such as in hole applications, band and strip placements, in particular in paddy soils throughout China. One of the key advantages of these application methods is a reduction of N loss through nitrification-coupled denitrification^16^ and ammonia (NH_3_) volatilisation^17^. When urea granules are applied into paddy soil in a concentrated manner, N patches establish rapidly, resulting in significant N heterogeneity in paddy soils, which are further accentuated by spatiotemporal fluctuations. The microzone around such intensively applied N fertiliser displays significant variations in ion concentrations, pH, chemical N transformation rates, and rice root behavior, which cannot be deduced from analysis of the bulk soil.

A paddy field has a complex three-dimensional structure within N patches that are formed by concentrated fertilisation. Naturally, when N fertiliser is applied, much higher N concentrations will be established at the fertilisation site and the surrounding microcosm than in unfertilised locations (i.e., the bulk soil), in particular during the time periods immediately following applications. Geostatistical descriptions of soil-N heterogeneity patterns for various ecosystems are abundant in the literature, but accounts on agricultural soils are rare^2^,^8^. Soil-N heterogeneity in association with N fertilisation using concentrated fertilisation in agricultural systems appears to be much more pronounced than that seen in natural ecosystem. Root proliferation into nutrient-rich zones or patches is considered by some as a foraging response to this heterogeneous nature of the nutrient environment, which depends on the growth rate of the plant, the geometry of the root system, and the size and strength of the patch encountered^18^. However, little is known about the extent to which N nutrient patches vary within themselves and the extent to which they impact surrounding soil and pivotal N-cycling processes such as nitrification.

Nitrification, which is performed by ammonia-oxidising bacteria (AOB)^19^ or ammonia-oxidising archaea (AOA)^20^,^21^,^22^,^23^ converting NH_4_^+^ to nitrite (NO_2_^−^), and then by nitrite-oxidising bacteria (NOB)^24^ converting the latter to nitrate (NO_3_^−^), is a key process in the global N cycle. Therefore, nitrification is critical to the supply of plant-available N in rice fields as well as to the overall balance of NH_4_^+^ and NO_3_^− ^25. Regardless of whether AOB, AOA, or NOB are all strict aerobic chemolithoautotrophic microorganisms, the microbial N transformations can only occur in the presence of oxygen (O_2_) and, thus, would be restricted to the upper few millimeters of flooded soils where O_2_ is measurable^26^. In our recent studies, the O_2_ dissolved in the rhizosphere soil was found to be significantly improved by radial O_2_ loss (ROL) from rice roots, promoting rhizospheric nitrification^27^. Duan *et al.*^28^ and Zhao *et al.*^29^ found that partial replacement of NH_4_^+^ with NO_3_^−^ could greatly improve the growth, yield, and N acquisition of rice. They speculated that the increased uptake of NH_4_^+^ was promoted by NO_3_^−^. Clearly, the NO_3_^−^ that is produced in nitrification at the root surface and within the rhizosphere in soils is very important for rice N nutrition^30^. The relationship between rhizosphere nitrification and NUE in rice was demonstrated in our previous study^31^.

N concentrations within fertiliser patches can be expected to be manifold higher than in the bulk soil. However, it remains unanswered what scale and magnitude such differences might assume in waterlogged paddy soils and how the spatial variations of NH_4_^+^, NO_3_^−^, nitrification activity, pH, O_2_ and their temporal changes contribute to the spatial variation of the total inorganic N pool. In this study, two paddy soils were selected to examine these questions. The selected soils have the following characteristics: (1) they were collected from two representative rice production areas in subtropical China: Yingtan city, Jiangxi Province (YT paddy soil) and Qianjiang city, Hubei Province (QJ paddy soil); (2) YT paddy soil was derived from Quaternary red clay with a low overall pH value, and QJ paddy soil was derived from river alluvium with a high overall pH value; (3) both soils are subjected to similar fertility levels and tillage practices; (4) the average annual yields (obtained by double-cropping of rice) in Yingtan are significantly lower than those in Qianjiang. To obtain precise results for soil pH and O_2_, microelectrodes were used in our experiments. Microelectrodes were produced with a small-enough tip diameter (ø = 25 μm, Unisense, Aarhus, Denmark) to facilitate ready insertion into soil and plant tissues without damage, such as are routinely used in *in situ* measurements in plant physiology, environmental science, and related fields^32^. Hence, the present study was designed to measure and analyze, by path analysis (see methods), soil NH_4_^+^, NO_3_^−^, pH, and O_2_ within N patches in paddy soils with the goal of substantially improving our understanding of the microbial nitrification process that is pivotal to rice N nutrition and of the factors that control it^33^ within the heterogeneous reality of soil-N chemistry.

## Results

### Spatiotemporal fluctuations of ammonium and nitrate concentrations in nitrogen patches

**Figure 1:**
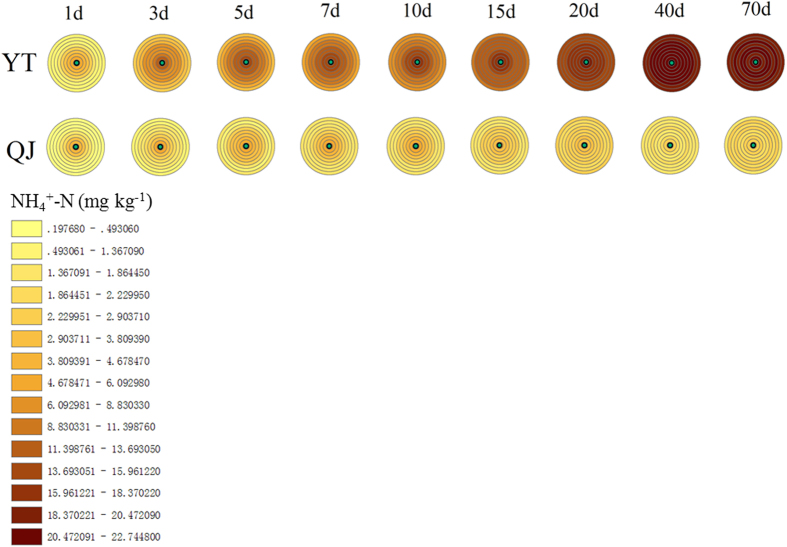
Distribution of NH_4_^+^ concentrations in Yingtan (YT) and Qianjiang (QJ) paddy soils. The concentric circles in the upper row represent YT paddy soil, and the concentric circles in the row below represent QJ paddy soil. The concentrations measured at different distance from the fertilisation site (the circles in the concentric circles, from inside to out, represent 0, 2, 4, 6, 8, 10, 20, 30 and 40 mm distance from the fertilisation zone, respectively) at different sampling dates (the concentric circles left-to-right represent 1, 3, 5, 7, 10, 15 20, 40 and 70 days after fertilisation, respectively). Mean values are shown for a sample size of three replicates.

It was obvious that the NH_4_^+^ concentrations strongly decreased with increasing distance from the N fertilisation site (Fig. 1). For example, the NH_4_^+^-N concentrations of YT and QJ at the fertilisation site were 2.66 and 3.92 times those measured at a distance of 40 mm away from the fertilisation site, at 7 d after fertilisation, respectively. The range of the N patch became more extensive with time, and disappeared at 40 d after urea application in both paddy soils (Fig. 1). The temporal variations of NH_4_^+^ concentrations in N patches differed between the paddy soils derived from different parent material. The NH_4_^+^ concentrations in the fertiliser patch of YT and QJ increased and decreased with incubation time, respectively, and became constant by 40 d following N application (Fig. 1), except for NH_4_^+^ concentrations near the N-application sites in QJ paddy soil, which exhibited an initial increase followed by a decrease within 40 d after N application (Fig. 1). The NH_4_^+^ concentrations at a far distance from the N-fertilisation site (>20 mm away) and those in control (CK) without fertiliser application, increased with time in both paddy soils within 40 d following N application (Supplementary Fig. S1). Both paddy soils differed significantly in NH_4_^+^-N concentrations, with an average of ~20 mg kg^−1^ in YT soil and ~2 mg kg^−1^ in QJ soil, respectively. Compared with CK, N fertilisation significantly increased the NH_4_^+^ concentration in the paddy soil (Supplementary Fig. S1).

**Figure 2:**
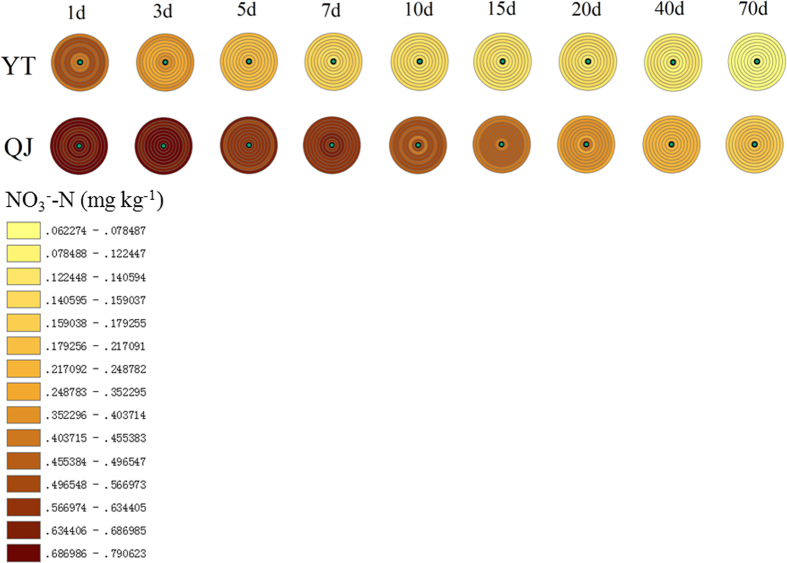
Distribution of NO_3_^−^ concentrations in Yingtan (YT) and Qianjiang (QJ) paddy soils. The concentric circles in the upper row represent YT paddy soil, and the concentric circles in the row below represent QJ paddy soil. The concentrations measured at different distance from the fertilisation site (the circles in the concentric circles, from inside to out, represent 0, 2, 4, 6, 8, 10, 20, 30 and 40 mm distance from the fertilisation zone, respectively) at different sampling dates (the concentric circles left-to-right represent 1, 3, 5, 7, 10, 15 20, 40 and 70 days after fertilisation, respectively). Mean values are shown for a sample size of three replicates.

**Figure 3:**
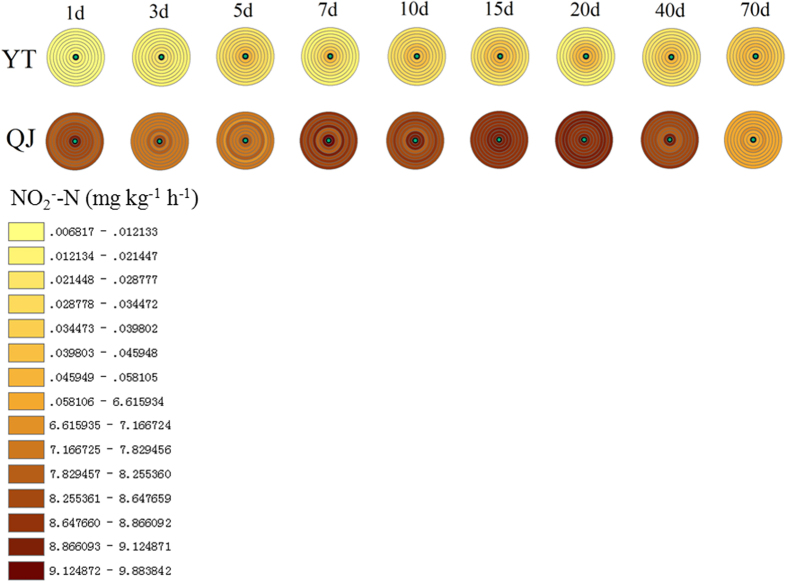
Distribution of short-term nitrification activity in Yingtan (YT) and Qianjiang (QJ) paddy soils. The concentric circles in the upper row represent YT paddy soil, and the concentric circles in the row below represent QJ paddy soil. The nitrification activities measured at different distance from the fertilisation site (the circles in the concentric diagrams, from inside to out, represent 0, 2, 4, 6, 8, 10, 20, 30 and 40 mm away from the fertilisation zone, respectively) at different sampling dates (the concentric circles left-to-right represent 1, 3, 5, 7, 10, 15 20, 40 and 70 days after fertilisation, respectively). Mean values are shown for a sample size of three replicates.

Compared with the spatiotemporal variations in NH_4_^+^, there were no significant spatiotemporal NO_3_^−^ patch phenomena in the paddy soils tested (Fig. 2). When urea was applied to the waterlogged soils, NO_3_^−^ concentrations showed almost no concentration gradients in terms of spatial distribution. The NO_3_^−^ concentrations in the N-application sites at the beginning of N fertilisation (1 d after fertilisation in YT soil) were significantly lower than those in controls without fertilisation (Fig. 2). The NO_3_^−^ concentrations decreased with time, while the pattern of decrease differed between the two paddy soils. The NO_3_^−^ concentration in YT paddy soil peaked at 1 d after N fertilisation, then decreased slightly at 3 d after N fertilisation, and became stable at 5 d after N fertilisation (Fig. 2). The NO_3_^−^ concentration in QJ paddy soil showed a gradual decline during the incubation period (Fig. 2). The maximal NO_3_^−^ concentration of the QJ was significantly higher than that measured in the YT soil (Fig. 2), which might be due to the higher nitrification activity in QJ than in YT soil (Fig. 3). N fertilisation increased the NO_3_^−^ concentration in YT soil (Supplementary Fig. S2). Within 7 d after N fertilisation, there was no significant effect of N fertilisation on the increase in NO_3_^−^ concentration, while the pattern of increase became obvious after that period in the QJ treatment (Supplementary Fig. S2).

### Spatiotemporal fluctuations in nitrification activity in nitrogen patches

Fertiliser patch phenomena for nitrification were significant in paddy soil derived from red soil (YT), while they seemed not obvious in paddy soil derived from river alluvium (QJ). Nitrification activities within the N patches were significantly higher than those in the bulk soil, and they decreased with distance from the fertilisation site in YT soil, (Fig. 3), while there was no difference among the various sites sampled in QJ soil (Fig. 3). In YT soil, N-patch nitrification activity and patch range increased slowly over time during the first 7 d, and a rate of 0.05 mg kg^−1^ h^−1^ was maintained during the period of 40 d after fertilisation, after which the N-patch effect disappeared, at 70 d after fertilisation (Fig. 3). The nitrification activity measured in QJ soil showed almost no variance with time, with values of ~8.5 mg kg^−1^ h^−1^ during the first 40 d, but decreased significantly at the last sampling time (Fig. 3). An unexpected observation was that the nitrification activity measured in QJ soil was nearly 200 times that measured in the YT soil. Compared to control, N fertilisation significantly increased soil nitrification (Supplementary Fig. S3).

### Spatial pH fluctuations in nitrogen patches

**Figure 4:**
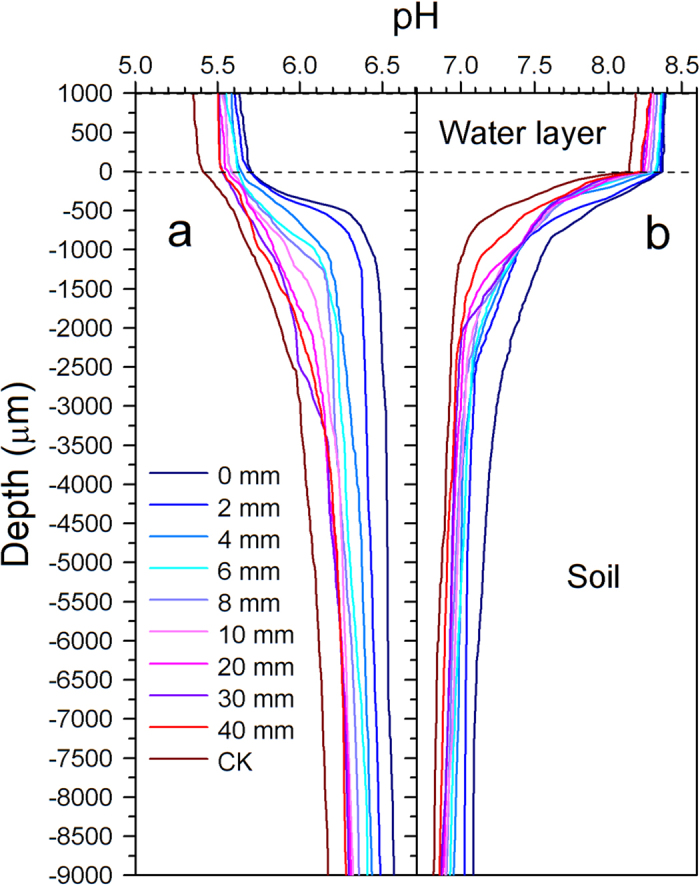
pH profiles in (**a**) Yingtan (YT) and (**b**) Qianjiang (QJ) paddy soils measured at different distances from the fertilisation site at 40 d after fertilisation. A 25-μm-diameter pH microelectrode was used (pH 25, Unisense, Aarhus, Denmark). CK, without N fertilisation. The ordinate represents the depth of the water layer (0–1,000 μm) and soil (0–9,000 μm), and the tick label of “0” shows the water-soil interface. The pH profile was determined in the right and left compartments of each box and in the center at different distances from the nylon nets in each treatment. Profile results in repeats were so similar that a representative result is shown.

The pH values measured in the water layer slightly decreased with horizontal distance from the fertilisation sites in both soil types, while those in YT were much lower than in QJ, with ~5.5 and 8.3, respectively (Fig. 4). When the microelectrode tip touched the water-soil interface, the pH value measured increased sharply in YT soil, while declining sharply in QJ soil. pH values varied greatly with soil depth initially but became constant at 1.0–3.5 mm depth, approaching neutrality (Fig. 4). Soil pH values decreased with distance from the fertilisation sites, and pH values measured in the water layer and in soil in the CK were significantly lower than those in the fertilisation treatments, indicating that urea fertilisation can enhance the water-layer and soil pH in waterlogged paddy soil by about 0.5 to 1 pH units. Urea fertilisation seemed to impact soil pH more significantly in paddy soil derived from red soil (YT) than in paddy soil derived from river alluvium (QJ), i.e. the fertiliser patch phenomenon vis-à-vis soil pH is more pronounced in paddy soil derived from red soil. Overall, soil pH values were higher in QJ than in YT (Fig. 4). For example, pH values were 6.4 and 7.6 at a depth of 0.5 mm from the soil surface, and 6.5 and 7.2 at a depth of 5 mm in the YT and QJ, respectively (Fig. 4).

### Spatial fluctuations in oxygen concentration in nitrogen patches

**Figure 5:**
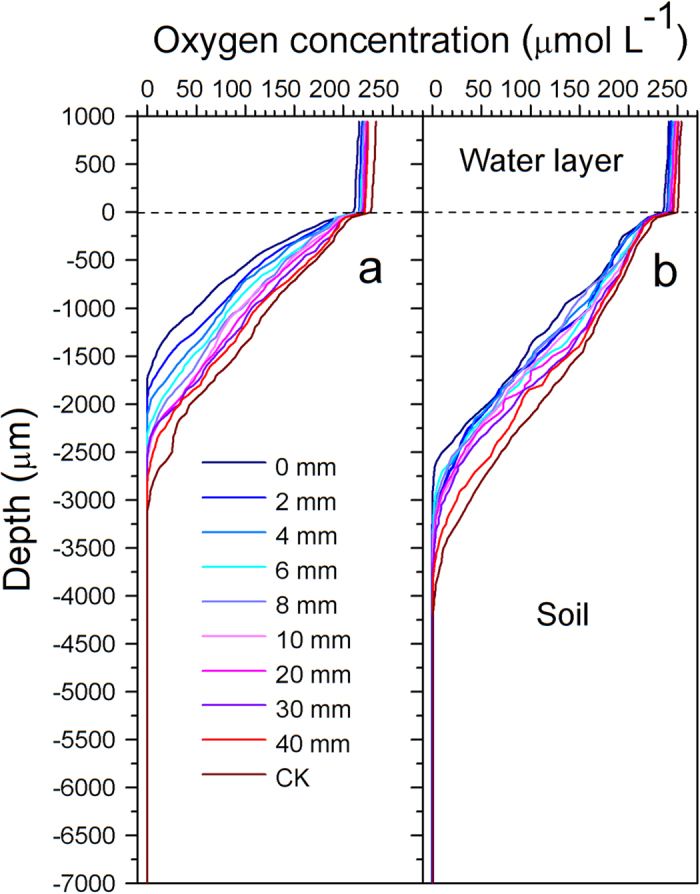
Oxygen concentration profiles in (**a**) Yingtan (YT) and (**b**) Qianjiang (QJ) paddy soils measured at different distances from the fertilisation site at 40 d after fertilisation. A 25-μm-diameter O_2_ microelectrode was used (OXY25, Unisense, Aarhus, Denmark). CK, without N fertilisation. The ordinate represents the depth of the water layer (0–1,000 μm) and soil (0–7,000 μm), and the tick label of “0” shows the water-soil interface. The O_2_ profile was determined in the right and left compartments of each box and in the center at different distances from the nylon nets in each treatment. Profile results in repeats were so similar that a representative result is shown.

Oxygen dissolved in the water layer remained constant near 220 and 244 μmol L^−1^ in the YT and QJ soils, respectively (Fig. 5). O_2_ concentration declined sharply in the water-soil interface to 5–10% compared with that in the water layer. With increasing soil depth, O_2_ concentrations decreased rapidly and smoothly and became undetectable within 1.7–4.0 mm depth below the soil surface (Fig. 5). The soil depth at which dissolved O_2_ concentration became undetectable showed a ranking of QJ > YT, and that depth was greater in controls than in fertilisation treatments. The O_2_ concentration in the fertilisation sites, when assessed horizontally, consistently showed minimal levels and increased with distance away from the fertilisation sites in both treatments, and the CK showed maximal O_2_ concentrations compared to the fertilisation treatments. Urea fertilisation decreased the soil O_2_ concentration by about 50% compared with CK (Fig. 5). The O_2_ concentrations in both water layers and in soil ranked QJ > YT.

### Major soil properties affecting nitrification activity

Simple correlation coefficients (*r*) between NH_4_^+^, NO_3_^−^, pH_water_ (water pH), pH_soil_ (soil pH), O_2water_ (water O_2_) and O_2soil_ (soil O_2_) are presented for comparison with path analysis results. The uncorrelated residual value (*U*) was low (0.077), while the coefficient of determination (*R*^*2*^) was high (0.994), indicating that the path analysis model could explain the majority (99.4%) of variation in soil nitrification (Table 1). Path analysis partitioned each r value into one direct effect and five indirect effects. Partitioning by path analysis showed significant direct effects by pH_water_ (*P*_*37*_ = 1.234, p < 0.001) and pH_soil_ (*P*_*37*_ = −0.154, p < 0.001) on nitrification. Furthermore, the direct effects of NH_4_^+^, NO_3_^−^, O_2water_, and O_2soil_ on nitrification were not significant (p > 0.05), while the simple correlation analysis revealed that NH_4_^+^, NO_3_^−^, pH_water_, pH_soil_, O_2water_, and O_2soil_ were all significantly correlated with nitrification (p < 0.001). Examination by path analysis revealed that pH_water_ had negative indirect path coefficients through NH_4_^+^ (*r13P37* = −1.220, p < 0.001), while it had positive indirect path coefficients through NO_3_^−^ (*r23P37* = 1.196, p < 0.001), pH_soil_ (*r34P37* = 1.141, p < 0.001), O_2water_ (*r35P37* = 1.183, p < 0.001), and O_2soil_ (*r36P37* = 1.098, p < 0.001).

## Discussion

Ammonium derived from the mineralisation of organic matter is transformed into NO_3_^−^ through the nitrification process. Both ions are presented in variable concentrations in different ecosystems, and frequently plants show preferences for one N form or another^34^,^35^. The fate over time of NH_4_^+^ and NO_3_^−^ concentrations in N patches in agricultural soils such as paddies had not been hitherto examined. Dramatic such changes were seen in our study, although the different origin and behavior of the two N sources in soils will influence spatial distribution^12^ (Figs 1 and 2).

In the paddy soils in our study, NH_4_^+^ was generated by urea hydrolysis, with additional contributions from soil organic N. Consequently, NH_4_^+^ concentrations inside the N patch were significantly higher than those in the bulk soil or in unfertilised soil (Fig. 1, Supplementary Fig. S1). NH_4_^+^ concentrations decreased with increasing distance from the N fertilisation site, and the range of the NH_4_^+^-N patch increased over time (Fig. 1). This result supports the notion that transport of NH_4_^+^ in soil occurs mainly by diffusion^33^. A large fraction of organic N was present in both paddy soils at the start of the experiment (see Methods). Because farming practices on the two paddy fields from which the soils derived are characterised by long-term inorganic and organic fertilisation in the rice season and manure application in the fallow (winter) season (reclamation greater than 50 years). The process of mineralisation of the organic N pool is an important contributor to the generation of inorganic N in the soil^31^. A comparison between the treatments and the controls allowed for calculations that revealed that less than 30% of NH_4_^+^ accumulated in the N patch derived from mineralisation before 10 d after fertilisation. Certainly the proportion of mineralised NH_4_^+^ increased with time. At the preliminary stage of fertilisation, NH_4_^+^ accumulation resulting from mineralisation accounted for a much smaller percentage. For example, the proportions of NH_4_^+^ derived from mineralisation were only about 3.5% at 1 d after fertilisation. Paddy soils collected from Taihu Region, one of the most ancient rice cultivation areas of China, were studied with 120-day anaerobic incubation at 25 and 35 °C without rice planting, and the organic N mineralisation ranged from 4.0% to 9.4% of total N (60–241 mg kg^−1^)^36^. Thus, NH_4_^+^ generation from organic N in both paddy soil types may be expected to be high. However, NH_4_^+^ concentrations in QJ were much lower than in YT (except in the fertilisation zone on the 1st day following fertilisation; see Fig. 1). Ammonium ions, loosely bound to water molecules, predominate in water at a pH value above 7.2. With increasing hydroxyl-ion concentrations in the water, ionised NH_4_^+^ increasingly converts to nonionised NH_3_, which increases the risk of NH_3_ volatilisation^37^. Because the pH values tested both in the water layer and soil of QJ were all higher than in YT, and the pH in the water layer was above 8.0 (Fig. 4). Therefore, NH_3_ generated from NH_4_^+^ in QJ paddy soil was volatilised more intensely. The unexpectedly high nitrification activities measured in the QJ paddy soil (more than 200 times those seen in the YT paddy soil, see Fig. 3) might lead to large quantities of NH_4_^+^ consumption as nitrification substrate, further accentuating NH_4_^+^ depletion (Fig. 1).

In contrast to most agricultural soils, where NO_3_^−^ is the predominant N form, the flooded conditions in paddy soil greatly restrain the microbial formation of NO_3_^−^, and therefore NH_4_^+^ is the main form of N available to young rice plants^38^. Indeed, NO_3_^−^ concentrations measured in both soils in our study were two orders of magnitude lower than NH_4_^+^ concentrations (Figs 1 and 2). The NO_3_^−^ diffusion rate in flooded lowland soils has been determined to be 1.33 cm^2^ day^−1^, which is approximately 7 times faster than NH_4_^+^ diffusion^39^. Therefore, diffusion is expected to play a more important role for NO_3_^−^ distribution in flooded paddy soil than for that of NH_4_^+^, which is relatively even (Fig. 2). Since NO_3_^−^ is an anion, not significantly adsorbed by soil colloids, it tends to diffuse rapidly to other parts of the soil, unlike NH_4_^+^, which is readily adsorbed by negatively charged soil colloids. However, NO_3_^−^ dynamics in association with NO_3_^−^-N patches are more complex. Despite nitrification, nitrifier denitrification^40^ (the pathway of nitrification in which NH_3_ is oxidised to NO_2_^−^ followed by the reduction of NO_2_^−^ to nitric oxide (NO), nitrous oxide (N_2_O), and molecular nitrogen (N_2_)) and NO_3_^−^ reduction to NH_4_^+^ (DNRA)^41^ would also influence spatiotemporal dynamics of NO_3_^−^ in paddy soils. We also note that, in our experiment, the observed NO_3_^−^ decreases and NH_4_^+^ accumulation in the YT soil over time (Figs 1 and 2), could also be attributable to DNRA.

Given the stoichiometry of nitrification, 2 mol of O_2_ and 1 mol of NH_4_^+^ are consumed per 1 mol of NO_3_^−^ formed^42^. Once formed, NO_3_^−^ may diffuse down into the reduced soil where it can be denitrified^43^. O_2_ and NH_4_^+^ depletion over time, and denitrification activity in the more anaerobic soil strata, all led to a decrease of NO_3_^−^ concentrations with time (Fig. 2), even though nitrification activities themselves in both soils did not show distinct decreases over time (Fig. 3). Because rice roots can efficiently absorb NO_3_^− ^44, the denitrification substrate is accordingly removed rapidly in the field, reducing denitrification in the rhizosphere. In some sense, therefore, rice planting can decrease N loss by denitrification compared to fallow soil. Paddy soil without rice, such as examined in our study, would increase the risk of denitrification, and accordingly decrease the NO_3_^−^ dissolved in the paddy soil. Using the multiple ring buffer graphics in our experiments, the spatiotemporal fluctuations of soil NH_4_^+^ and NO_3_^−^ concentrations within N patches could be shown intuitively (Figs 1 and 2). Also, the pattern of growth and decline in nitrification activity within N patches was well visualised (Fig. 3) by using this kind of graphic, and is presented here for the first time.

Unlike in other ecosystems, paddy soil is deficient in dissolved O_2_ because of the agricultural practice of regular flooding. The O_2_ is detected only in a millimeter-thick surface layer, leaving the bulk soil anoxic, restricting the activity of nitrifying microorganisms to this small zone^33^. The available O_2_ in flooded paddy soil is mainly derived from the micro-amounts of O_2_ dissolved in the soil and the O_2_ from irrigation water, and is enhanced only immediately within the rice rhizosphere by root-directed O_2_ transport and exudation through rice aerenchyma tissue^42^. In our study, although the N patch seemed to be dissolved by 40 d following urea application in both paddy soils (Fig. 1), O_2_ concentrations and pH still revealed different horizontal distribution characteristics between the N-patch area and the bulk soil (Figs 4 and 5).

Urea fertilisation could increase the water-layer and soil pH in waterlogged paddy fields by about 0.5–1 pH units (Fig. 4). Urea fertilisation seemed to produce a more significant change in soil pH in acid paddy soil (YT) than in alkaline paddy soil (QJ). Volatilisation losses can occur from acid as well as alkaline soils due to high pH and NH_4_^+^ enrichment at the microsite where urea granules dissolve and hydrolyze. Acidification of the fertiliser microsite is a mechanism by which NH_3_ volatilisation may be reduced^45^. In our experiment, *in situ* results showed that pH became constant at ~1.0–3.5 mm depth below the soil surface and approached neutrality in both soils tested (Fig. 4). However, these results might be different if rice planting were to occur. The pH of rice paddy soils following two weeks of flooding usually ranges from 6.5–7.0, although some reports have shown pH to remain as low as 5.0 or as high as 8.0^45^. Fortunately, the rice root system (mainly composed of adventitious root at this stage of development) tends to be concentrated in 0–5-cm depth in paddy soil^27^, i.e. in the neutral-pH zone, which allows rice roots to avoid damage from relatively high or low pH. Like pH, O_2_ concentration also showed fertiliser-patch phenomena. Urea fertilisation decreased the soil O_2_ concentration to about 50% compared with the unfertilised treatment at 40 d after fertilisation in the waterlogged condition (Fig. 5). Aerobic processes such as nitrification are intensified within the N patch, leading to soil oxygen depletion there.

The availabilities of NH_4_^+^ and O_2_ are key factors determining the rate of nitrification^25^. Although the NH_4_^+^ concentration in the QJ paddy soil was significantly lower than in the YT soil, the relatively higher pH (Fig. 4) and O_2_ concentration (Fig. 5) promoted nitrification (Fig. 3). Due to the higher amounts of O_2_ consumed in the degradation of organic matter and in the oxidation of reduced compounds^46^, YT soil displayed more severe O_2_ depletion, given its higher organic matter content (see Methods). In addition, YT soil had relatively lower porosity (because of its higher clay content, see Methods), which led to less O_2_ dissolution (Fig. 5).

Natural factors, such as soil parent materials, topography, climate, as well as human factors, such as reclamation time and history of fertilisation, are all important factors influencing the soil-formation process in paddy soils^47^. Due to differences in soil formation, soil properties vary considerably among soil types^48^, and these, in turn, influence soil N-transformation processes, in particular nitrification and denitrification. However, soil properties are often strongly interrelated, making it challenging to establish a causal relationship between soil properties and nitrification activity if a simple correlation analysis is used. By contrast, path analysis is a statistical technique that distinguishes correlation and causation by partitioning correlation into direct and indirect effects^49^. The direct effect represents the direct contribution of a predictor variable (e.g., soil properties) to a response variable (e.g., nitrification activity). The indirect effect represents the contribution of a predictor variable to a response variable via another predictor variable. Therefore, path analysis can provide insight into the relative contribution of causal relationships and the direction of a causal path. Path analysis has been used extensively in agronomic studies, such as in examining the relationships between soil properties and phosphorus sorption capacity^50^ or the adsorption of heavy metals^51^. However, to the best of our knowledge, path analysis has not been hitherto employed to examine the relationships between soil properties and nitrification activity.

In our experiments, soil O_2_ concentration and pH might be the principal factors affecting nitrification as nitrification substrate was not limiting, and the manifestations of these determinants were different between the soil types. Path analysis confirmed the main contribution by pH, especially water-layer pH, which played the most significant role (*P*_*37*_ = 1.234, p < 0.001) in nitrification, and the second contributor was soil pH (*P*_*37*_ = −0.154, p < 0.001; see Table 2). Although simple correlation analysis revealed that O_2_ dissolved in the water layer and soil were both significantly correlated with nitrification (p < 0.001), path analysis revealed the correlation mainly derived from the strong effect of water-layer pH on O_2_ dissolved in the water layer (*r35P37* = 1.183, p < 0.001) and the soil profile (*r36P37* = 1.098, p < 0.001; Table 2); path analysis partitions correlations into direct and indirect effects and distinguishes between correlation and causation^49^. It should be noted that the use of mean values for O_2_ measured in identical vertical profiles, necessitated by the limitations in the soil slice collection method, might lead to small underestimations of nitrification rates, as the penetration depth of O_2_ into the soil strongly affects nitrification and is typically limited to the uppermost few millimeters of soil. However, as the same procedure was applied throughout, we posit that the data between our treatments are directly comparable, and the correlations and trends observed hold even though a finer resolution for soil sample collection was not technically feasible.

Generally speaking, N patches derived from concentrated fertilisation shows a dramatically changing spatio-temporal pattern in paddy-soil environments, and nitrification displays colourful performance at different stages. The rice plant once planted into paddy soil is expected to affect the nitrification process, while its behavior, in turn, would be greatly affected by the patch. Although the results obtained from fallow soil are not in themselves sufficient to understand the behavior of the paddy field situation that includes the rice crop, and a diverse mesofauna and macrofauna, study of the simplified soil system represents an important first step and is essential to the understanding of the detailed behavior of nitrification within N patches on account of soil properties. Poising N-fertiliser applications efficiently and without negative environmental consequences will require a thorough understanding of patch phenomena in agricultural soils. The dynamics within N patches in paddy soil in the presence of rice should be the subject of dedicated future research efforts.

## Methods

### Description of the paddy soil collected sites

The paddy soils were collected from two representative rice production areas in subtropical China, Yingtan city (YT paddy soil, 28°15′20″ N, 116°55′30″ E), Jiangxi Province and Qianjiang city (QJ paddy soil, 30° 15′ 36″ N, 112° 31′ 48″ E), Hubei Province. The detailed properties of both paddy soils are shown in Table 2.

### Soil incubation and sampling

An organic glass incubation box was divided into three compartments with two nylon nets (30 μm mesh size) where fertiliser-soil mixture was placed in the middle of the box and soil without fertiliser was in the two side compartments. The mesh of the nylon net was fine enough to allow penetration of water and nutrient elements. Design and specifications of this incubation box were as displayed in the rhizobox diagram^14^ (detail of the incubation box see Supplementary Fig. S4).

Each incubation box was filled with 600 g of paddy soil (air-dried, ground and sieved through 0.85 mm, which explicitly excludes soil mesofauna and macrofauna). In the middle compartment, 100 g soil was mixed thoroughly with urea (equivalent to 120 mg N kg^−1^) and KH_2_PO_4_ (93 mg kg^−1^). The two side compartments were simultaneously filled with 250 g soil without fertiliser. For control (CK) experiments, boxes were filled with 600 g paddy soil without urea but with KH_2_PO_4_ (93 mg kg^−1^) in the middle compartment. A depth of 1 cm of surface water was maintained by adding deionised water every morning and evening throughout the experimental period. All boxes were incubated in a constant incubator at 25 °C and with a 16 h photoperiod.

To avoid water layer interference with the chemical determinations, paddy soils were not watered in the evening of the day prior to the sampling date, to maintain a thin (1–2 mm) water layer. The advantage of this approach is that the soil is easy to slice and NO_3_^−^ concentrations can be determined more effectively. However, the disadvantage of this approach is that it imperceptibly stimulates nitrification and results in overestimates of the soil NO_3_^−^ concentration. Each sampling occurred at 7:00 am, and each treatment sample was kept in −20 °C for 2 h to rigidify the paddy soil for subsequent slicing; soil samples were then collected at distances of 0, 2, 4, 6, 8, 10, 20, 30 and 40 mm distance from the fertilisation zone, respectively, without vertical profiles (unlike the *in-situ* measurement of soil pH and O_2_ concentration), for logistical reasons, although vertical redox gradients in submerged soils are also expected to influence the distribution of NH_4_^+^ and NO_3_^− ^43. The soil samples collected from the right and left compartments at an identical horizontal distance were mixed together for the assessments of mineral N and nitrification activity at 1, 3, 5, 7, 10, 15 20, 40 and 70 days after fertilisation, with three replicates.

### Mineral N assay

Fresh soil samples were extracted with 2 mol l^−1^ KCl (soil: solution ratio 1:10), and extracts were measured for NH_4_^+^ and NO_3_^−^ by a continuous- flow auto- analyzer (model Autoanalyzer 3, Bran + Luebbe, Hamburg, Germany). In the preliminary experiment, NO_3_^−^ concentration in flooded soil was so low as to be undetectable using the original extracts. Thus, subsequently, extracts were concentrated 10 times prior to determination.

### Short-term nitrification activity assay

Short-term estimations are usually used in assays of nitrification activity. The principle is based on the determination of NO_2_^−^ after the incubation of soil samples with NaClO_3_ (an inhibitor of NO_2_^−^ oxidation) in the absence of NH_4_^+^ for 24 h at 25 °C^52^. The procedure is briefly described as follows. Each soil sample (5 g of moist soil) was shaken with 2.5 mL of NaClO_3_ (75 mmol L^−1^) at 170 rpm on an rotary shaker for 30 min and then incubated for a further 24 h at 25 °C in order to prevent NO_2_^−^ conversion into NO_3_^−^. After incubation, NO_2_^−^ was extracted from soil samples into a total volume of 15 mL using two solutions (first, 5 mL deionised H_2_O, and then 10 mL of 2 mol L^−1^ KCl) by shaking at a 170 rpm speed for 30 min on an rotary shaker as described earlier. The contents were mixed thoroughly and immediately filtered. Five mL of the clear filtrate was pipetted into glass test tubes, followed by 3 mL of buffer (0.19 mol L^−1^ NH_4_Cl, pH 8.5) and 2 mL of the reagent (dissolve 2 g of sulphanilamide and 0.1 g naphthyl-diethylene-diammonium chloride in 150 ml distilled water and 20 ml phosphoric acid were added. After cooling, dilution to 200 ml with distilled water) for NO_2_^−^ determination. The contents were again vigorously shaken and allowed to stand for 15 min at room temperature. Finally, the colour intensity was measured at 520 nm. For control measurements, soil samples were extracted as previously described after incubation with NaClO_3_ at −20 °C. The determination was repeated three times using four incubation boxes (three for the samples and one for the control). Pre-experiments using the paddy soil tested (waterlogged for 40 d) to monitor the kinetics of NH_3_ oxidation in the first 24 hours were performed, showing that NO_2_^−^ was produced linearly over time (p < 0.05). Short-term nitrification activity was expressed as the production of NO_2_^−^-N per unit time and calculated according to equation (1):


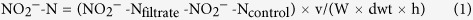


where NO_2_^−^-N is the production of NO_2_^−^-N per unit time (mg kg^−1^ h^−1^), NO_2_^−^-N_filtrate_ is the determination of NO_2_^−^-N after the incubation of soil samples with NaClO_3_ for 24 h at 25 °C (mg l^−1^), NO_2_^−^-N_control_ is the determination of NO_2_^−^-N after the incubation of soil samples with NaClO_3_ for 24 h at −20 °C (mg l^−1^), dwt is the dry weight of 1 g moist soil used, W is the weight of the soil used (g), v is the total volume of solutions added to soil sample in the assay (ml), t is the incubation time (h).

### *In situ* measurement of soil pH and oxygen concentrations

Incubation boxes, in three replicates, were also used for the *in-situ* measurement of soil pH and O_2_ concentrations at 40 d after fertilisation. The incubation condition was as described above, except that in boxes for *in-situ* measurements, a water layer of 1 cm was always maintained.

The pH microelectrode was a miniaturised pH glass electrode with an outer tip diameter of 25 μm^53^ (pH 25, Unisense, Aarhus, Denmark). The O_2_ microelectrode was also a miniaturised Clark-type O_2_ electrode with a guard cathode^54^ (OXY25, ø = 25 μm, Unisense, Aarhus, Denmark). The tips of both pH and O_2_ microelectrodes were highly fragile, so a micromanipulator (MM33-2, Unisense) with a motor controller (MC-232, Unisense) were used, with the micromanipulator fixed on the lab stand (LS18, Unisense) to avoid disturbance during the microelectrode movement. As measurements of pH values and O_2_ concentrations were very sensitive to the temperature, a thermometer was used to monitor soil temperature during the measurement. Both measurements for pH and O_2_ were conducted at 50-μm depth intervals, and the periods for “wait before measure” and “measure” were both set to 3 s. All the *in situ* measurements were performed in a zero-electrical-noise-interference laboratory, at 25 °C.

### Data analysis

All statistical analyses were performed using SPSS version 13.0, and one-way ANOVA with a homogeneity of variance test was performed, followed by an LSD test to check for quantitative differences between treatments. P < 0.05 was set as the significance cut-off.

**Figure 6:**
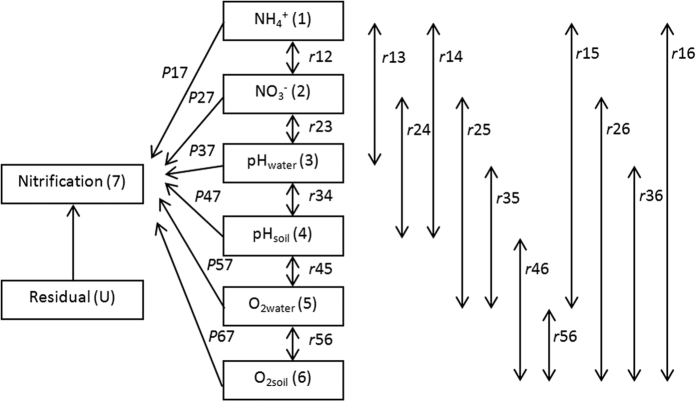
Path analysis diagram for the relationship between nitrification and soil properties. Path coefficients (*P*_ij_) of soil properties are represented by single-headed arrows while simple correlation coefficients (r_ij_) between variables are represented by double-headed arrows. Subscript designations for soil properties are identified numerically as follows: 1, NH_4_^+^ dissolved in soil (NH_4_^+^); 2, NO_3_^−^ dissolved in soil (NO_3_^−^); 3, pH value measured in the water layer (pH_water_); 4, pH value measured in the soil profile (pH_soil_); 5, O_2_ concentration measured in the water layer (O_2water_) and 6, O_2_ concentration measured in the soil profile (O_2soil_).

Path analysis was used to evaluate the relationships between nitrification and soil properties, including NH_4_^+^, NO_3_^−^, pH_water_ (water pH), pH_soil_ (soil pH), O_2water_ (water O_2_) and O_2soil_ (soil O_2_). Because NH_4_^+^, NO_3_^−^, and nitrification were measured at 1, 3, 5, 7, 10, 15 20, 40 and 70 days after fertilisation, respectively, without vertical determination, and O_2_ and pH were only measured at 40 d after fertilisation, the data for NH_4_^+^, NO_3_^−^, and nitrification at 40 d after fertilisation, as well as the mean values of the data of O_2_ and pH measured in the identical vertical profile (separated as water layer and soil vertical-profiles), were used for the path analyses. The direct effects of soil properties on nitrification are represented by single-headed arrows while coefficients of intercorrelation between soil properties are represented by double-headed arrows in Fig. 6. The direct effects of soil properties on nitrification are termed path coefficients and are standardised partial regression coefficients for each of the soil properties in the multiple linear regression against nitrification^55^. Indirect effects of soil properties on nitrification were calculated from the product of the simple correlation coefficient between soil properties and the path coefficient^56^. The correlation between nitrification and soil properties is the sum of the direct and indirect coefficients. In addition, an uncorrelated residue (U) was calculated for the model using the following equation (2):


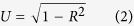


where R^2^ is the coefficient of determination in the multiple regression equation between nitrification and the six soil properties. Path analysis results were determined as described in the following equations:


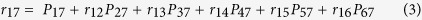



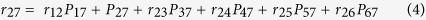



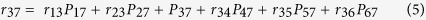



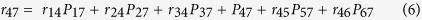



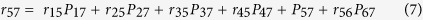



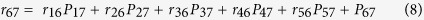


where r_ij_ is the simple correlation coefficient between nitrification and a soil property, P_ij_ is the path coefficient between nitrification and a soil property, and r_ij_P_ij_ is the indirect effect of a soil property on nitrification. Subscript designations are: 1, NH_4_^+^; 2, NO_3_^−^; 3, pH_water_; 4, pH_soil_; 5, O_2water_; and 6, O_2soil_. SPSS version 13.0 was used for statistical analysis.

## Additional Information

**How to cite this article**: Li, Y. *et al.* Microprofiling of nitrogen patches in paddy soil: Analysis of spatiotemporal nutrient heterogeneity at the microscale. *Sci. Rep.* **6**, 27064; doi: 10.1038/srep27064 (2016).

## Supplementary Material

Supplementary Information

## Figures and Tables

**Table 1:** Direct effects (diagonal, italics) and indirect effects (off diagonal) of soil properties on nitrification.

Variable	NH_4_^+^	NO_3_^−^	pH_water_	pH_soil_	O_2water_	O_2soil_	*r*	*R*^*2*^	*U*
NH_4_^+^	*0.111*	0.025	−1.220	0.135	−0.030	−0.009	−0.988***	0.994***	0.077
NO_3_^−^	−0.107	−*0.026*	1.196	−0.137	0.029	0.009	0.964***		
pH_water_	−0.110	−0.025	1.234***	−0.142	0.030	0.009	0.995***		
pH_soil_	−0.098	−0.023	1.141	−0.154***	0.026	0.007	0.900***		
O_2water_	−0.108	−0.025	1.183	−0.130	*0.031*	0.010	0.961***		
O_2soil_	−0.103	−0.023	1.098	−0.113	0.030	*0.010*	0.900***		

NH_4_^+^ (NH_4_^+^ dissolved in soil); NO_3_^−^ (NO_3_^−^ dissolved in soil); pH_water_ (pH value measured in the water layer); pH_soil_ (pH value measured in the soil profile); O_2water_ (O_2_ concentration measured in the water layer) and O_2soil_ (O_2_ concentration measured in the soil profile). ***significant at the level of p < 0.001, **significant at the level of p < 0.01, *significant at the level of p < 0.05.

**Table 2:** Properties of the two paddy soils used in the study.

Property	YT	QJ
pH (water:soil, 2.5:1)	5.02	8.05
Organic matter (g kg^−1^)	39.1	27.3
Total N (g kg^−1^)	1.74	1.49
C/N ratio	13.0	10.6
NH_4_^+^-N (mg kg^−1^)	2.27	1.46
NO_3_^−^-N (mg kg^−1^)	0.21	0.48
Clay (%)	20.9	12.7
Locality	Yingtan, Jiangxi prov.	Qianjiang, Hubei prov.
Reclamation year	>50	>50
Parent material	Quaternary red clay	River alluvium
Average yield of double cropping rice per year (kg ha^−1^)	10,000	12,500

Fertilisation strategy was in concentrated applications on both soils (see Methods).
