# Supplementary material for: Microprofiling of nitrogen patches in paddy soil: Analysis of spatiotemporal nutrient heterogeneity at the microscale
Source: Sci Rep. 2016 Jun 6;6:27064. doi: 10.1038/srep27064 (PMC4893627; doi:10.1038/srep27064)
Supplement: Supplementary Information [file srep27064-s1.pdf]

Title: Microprofiling of nitrogen patches in paddy soil: Analysis of spatiotemporal nutrient heterogeneity at the microscale

Authors: Yilin Li[1], Herbert J. Kronzucker[2] & Weiming Shi[1]*

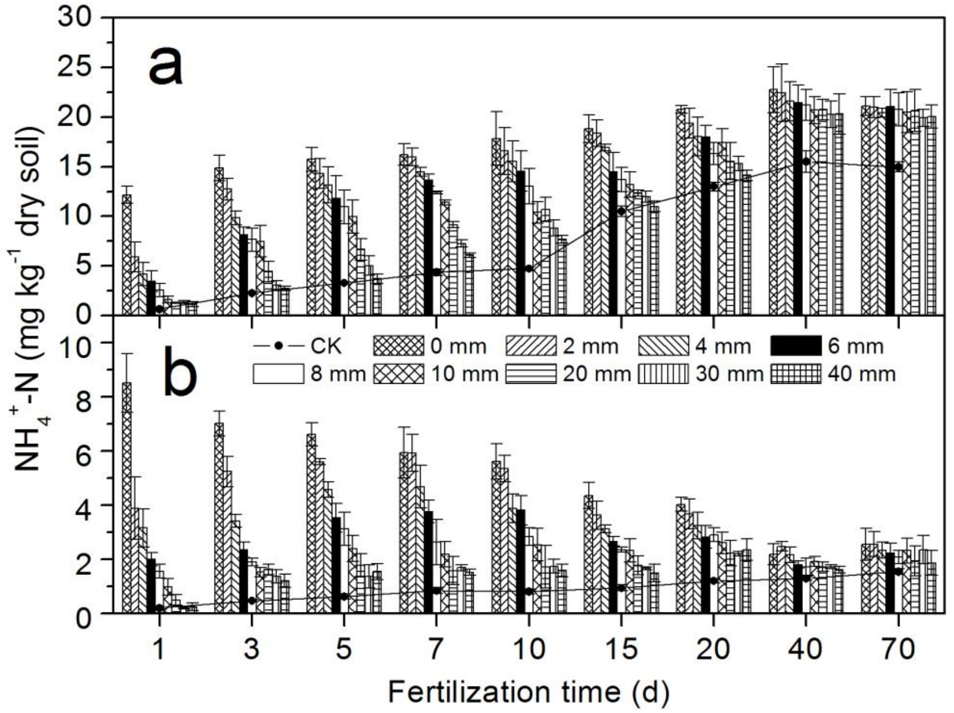

Figure S1. Distribution of NH_4^+ concentrations in (a) Yingtan (YT) and (b) Qianjiang (QJ) paddy soils measured at different distances from the fertilisation site on varying sampling dates. Mean values ± SD are shown for a sample size of three replicates. The curve indicates control (CK, without N fertilisation).

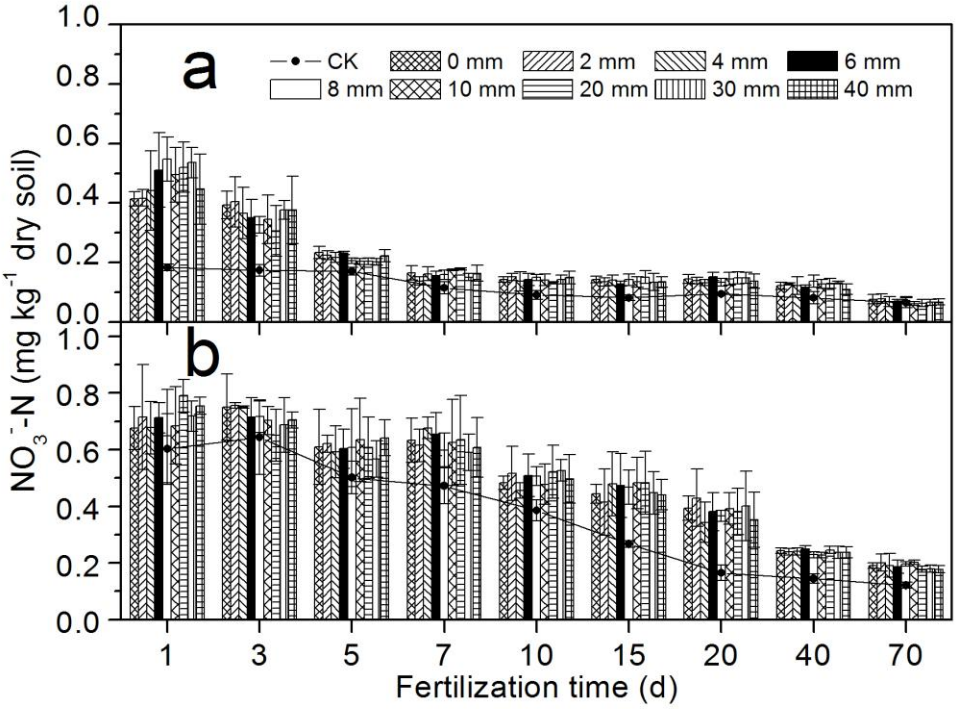

Figure S2. Distribution of NO_3^- concentrations in (a) Yingtan (YT) and (b) Qianjiang (QJ) paddy soils measured at different distances from the fertilisation site on varying sampling dates. Mean values ± SD are shown for a sample size of three replicates. The curve indicates control (CK, without N fertilisation).

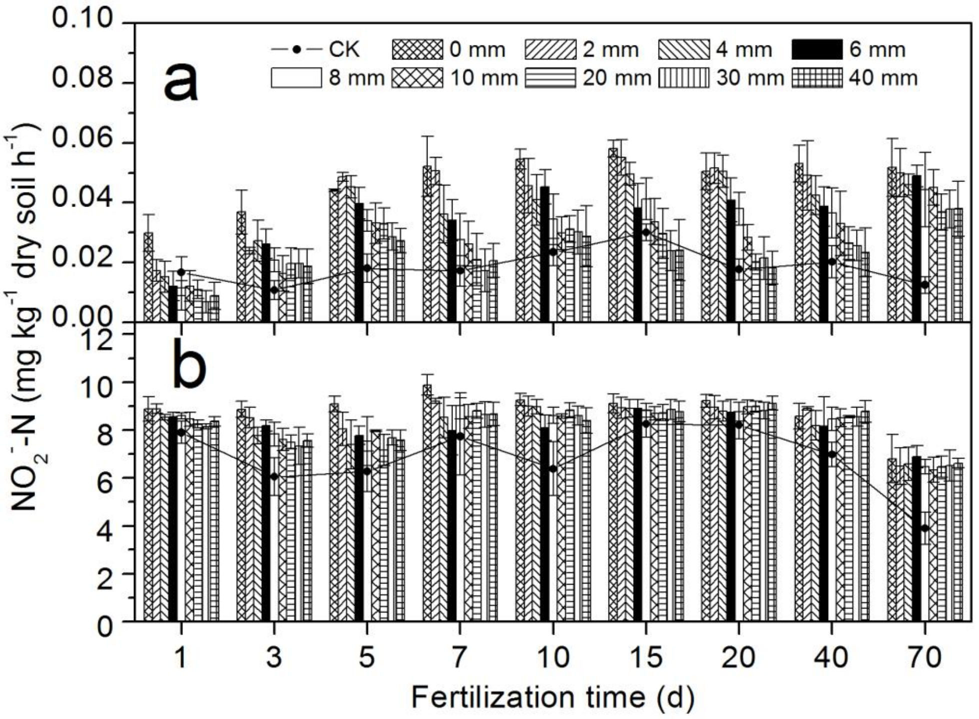

Figure S3. Distribution of short-term nitrification activities in (a) Yingtan (YT) and (b) Qianjiang (QJ) paddy soils measured at different distances from the fertilisation site on varying sampling dates. Mean values ± SD are shown for a sample size of three replicates. The curve indicates control (CK, without N fertilisation).

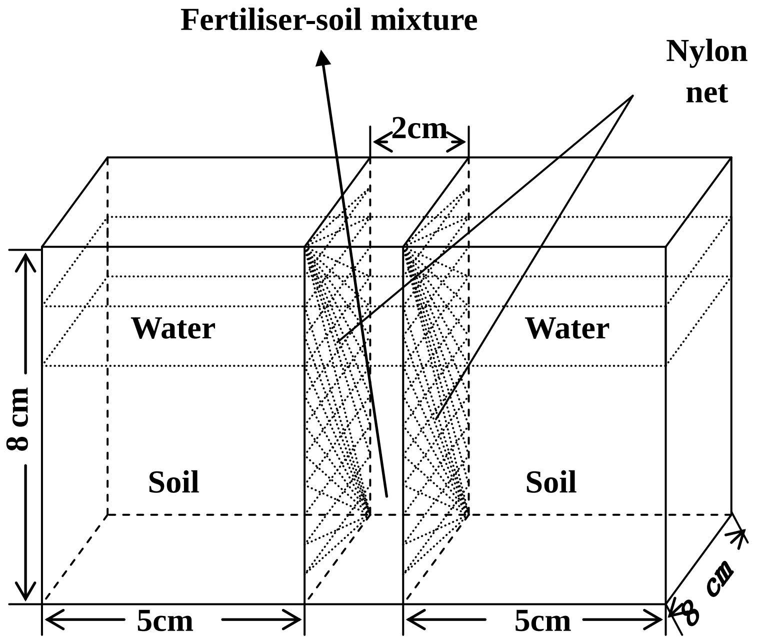

Figure S4. Diagram of the incubation box.
